# CD4^+^CD25^+^ T regulatory cells in renal transplantation

**DOI:** 10.3389/fimmu.2022.1017683

**Published:** 2022-11-08

**Authors:** Jason Cheung, Beata Zahorowska, Michael Suranyi, Jeffrey K. W. Wong, Jason Diep, Stephen T. Spicer, Nirupama D. Verma, Suzanne J. Hodgkinson, Bruce M. Hall

**Affiliations:** ^1^ Renal Unit, Liverpool Hospital, Sydney, NSW, Australia; ^2^ South Western Sydney Clinical School, University of New South Wales (UNSW), Sydney, NSW, Australia; ^3^ Immune Tolerance Laboratory, Ingham Institute for Applied Medical Research, University of New South Wales (UNSW), Sydney, NSW, Australia

**Keywords:** kidney transplantation, regulatory T cell, transplant tolerance, Treg, transplantation

## Abstract

The immune response to an allograft activates lymphocytes with the capacity to cause rejection. Activation of CD4^+^CD25^+^Foxp3^+^T regulatory cells (Treg) can down-regulate allograft rejection and can induce immune tolerance to the allograft. Treg represent <10% of peripheral CD4^+^T cells and do not markedly increase in tolerant hosts. CD4^+^CD25^+^Foxp3^+^T cells include both resting and activated Treg that can be distinguished by several markers, many of which are also expressed by effector T cells. More detailed characterization of Treg to identify increased activated antigen-specific Treg may allow reduction of non-specific immunosuppression. Natural thymus derived resting Treg (tTreg) are CD4^+^CD25^+^Foxp3^+^T cells and only partially inhibit alloantigen presenting cell activation of effector cells. Cytokines produced by activated effector cells activate these tTreg to more potent alloantigen-activated Treg that may promote a state of operational tolerance. Activated Treg can be distinguished by several molecules they are induced to express, or whose expression they have suppressed. These include CD45RA/RO, cytokine receptors, chemokine receptors that alter pathways of migration and transcription factors, cytokines and suppression mediating molecules. As the total Treg population does not increase in operational tolerance, it is the activated Treg which may be the most informative to monitor. Here we review the methods used to monitor peripheral Treg, the effect of immunosuppressive regimens on Treg, and correlations with clinical outcomes such as graft survival and rejection. Experimental therapies involving ex vivo Treg expansion and administration in renal transplantation are not reviewed.

## Introduction

Short-term renal transplant (RT) survival has significantly improved over the past few decades due to more potent immunosuppressive agents (1). Improving long-term RT survival remains a significant challenge due to chronic immune-mediated graft damage and side-effects of immunosuppression therapy (2). Inducing a state of “operational tolerance” where a graft remains rejection-free after withdrawal of immunosuppression is a sought-after ideal.

Several protocols aiming to induce transplant tolerance to a kidney allograft have been attempted (3–8). The original concept involved deleting clones of alloreactive lymphocytes, especially T cells (9). Injecting donor cells into neonatal rodents leads to thymic deletion of alloreactive cells (10). In adults depleted of lymphocytes, infusion of donor cells allows the thymus to delete the regenerating alloreactive cells (11). Whilst the main aim is to deplete clones reactive to the allograft, this is not essential to the induction of transplant tolerance, as recently reviewed (12). The other main mechanism is suppression or regulation. There are many different cell populations that can regulate immunity including CD4^+^ and CD8^+^T regulatory cells (Treg) (13), regulatory B cells (14–16), myeloid cells, and tolerizing antigen presenting cells. This review focuses on CD4^+^CD25^+^Foxp3^+^T regulatory cells (Treg).

The evidence, mainly from animal models of transplant tolerance, that T regulatory cells are major players in the induction and maintenance of transplant tolerance is reviewed here. We focus on the role of CD4^+^CD25^+^Foxp3^+^Treg which can mediate alloantigen specific tolerance, as reviewed (17) We examine the differences that distinguish naïve thymic derived CD4^+^CD25^+^Foxp3^+^Treg (tTreg) from antigen activated CD4^+^CD25^+^Foxp3^+^Treg, which mediate transplant tolerance. The latter population includes cells produced by activation of thymic derived naïve Treg, as reviewed below.

The review examines the many published studies that have attempted to monitor CD4^+^CD25^+^T cells, starting with studies on CD4^+^CD25^+^T cells. We examine the evolution in methods that attempt to identify the activated Treg most relevant to the mediation of alloantigen specific transplant tolerance.

The complexity of the very heterogenous CD4^+^CD25^+^T cell and CD4^+^CD25^+^Foxp3^+^T cell populations is described. We provide evidence that the most potent antigen specific Treg require continued activation by antigen and key cytokines produced by an ongoing effector T cell response. The rapid loss of function of antigen specific Treg ex vivo, has been underappreciated and has hampered attempts to study antigen specific Treg,

Finally, future directions that may better monitor activated Treg, which mediate transplant tolerance are discussed,

## Evidence that CD4^+^CD25^+^T cells can mediate transplant tolerance

Evidence for a key role of Treg in alloantigen specific tolerance is nearly solely derived from animal studies. In murine models, transplant tolerance can be induced by short-term non-specific immunosuppression without depletion of peripheral lymphoid cells, as recently reviewed (12). Lymphocytes from these tolerant hosts react to donor alloantigen *in vitro* (18) and in graft versus host assays (19), indicating there is no clonal deletion. These lymphocytes transfer tolerance and prevent normal cells mediating rejection *in vivo* (20–22). Transfer of tolerance is by CD4^+^T cells, not CD8^+^T cells or B cells (21). The CD4^+^T cells from tolerant hosts suppress co-transferred naïve CD4^+^T cells’ capacity to effect specific donor rejection. These CD4^+^T cells from tolerant hosts only suppress CD4^+^T cells reactive to specific donor.

Tolerant host CD4^+^T cells, that can transfer alloantigen specific tolerance, are not stable in culture however and regain the capacity to effect rejection (22, 23). Addition of both specific alloantigen and cytokines produced by activated lymphocytes to cultures of the suppressor CD4^+^T cells promotes the survival of inhibitory function (24–26). These studies identified that cytokines produced by activated T effector cells drive induction of antigen-specific Treg with many characteristics of the activated effector T cells (17, 27–31). These observations led us to examine for expression of CD25 on tolerance transferring CD4^+^T cells.

Murine studies of transplant tolerance first identified the alloantigen specific CD4^+^Treg (21) that express IL-2 receptor alpha (CD25), CD45(CD45RC) and MHC class II (22). The Summary in this manuscript describes the findings as *“The CD4^+^ suppressor was shown to be MRC Ox22^+^ (CD45R^+^), MRC Ox17^+^ (MHC class II), and MRC Ox39^+^ (CD25, IL-2-R)”* (22). This observation that CD25 is expressed by suppressor cells was unexpected as effector CD4^+^T cells activated by alloantigen are induced to express CD25 (32) and are the target of anti-rejection therapy with anti-CD25 monoclonal antibodies (mAb) (32, 33). Thus, both effector T cells and Treg depend on IL-2 for their activation and survival.

**Figure 1:**
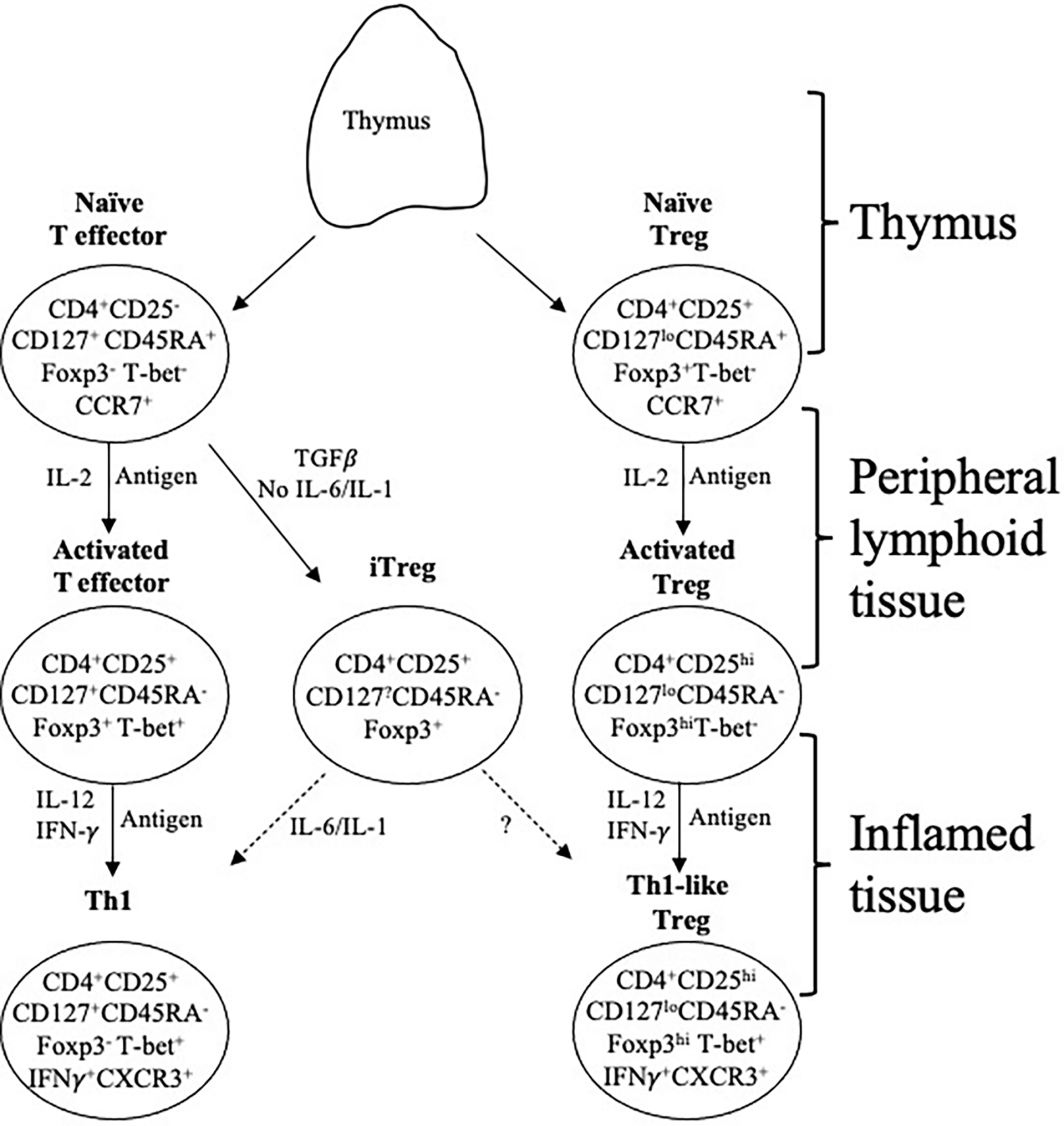
Effector lineage CD4^+^CD25^-^CD127^+^CD45RA^+^Foxp3^-^ and Treg. CD4^+^CD25^+^CD127^lo^CD45RA^+^Foxp3^+^ T cells are concurrently produced in the thymus and migrate from there to peripheral lymphoid tissue. They recirculate from lymphoid tissue to blood and back to lymphoid tissue, which is promoted by expression of CD62L and CCR7. When they recognize an antigen, they are activated and proliferate. In this process the effector lineage cells produce IL-2 and express IL-2R including CD25 (IL-2Rα chain). The IL-2 activates CD25 both on Treg and effector cells. In an immune response, naïve T cells (CD4^+^CD25^+^CD127^+^CD45RA^+^Foxp3^-^T-bet^-^CCR7^+^), in the presence of IL-2 and antigen, acquire CD25, Foxp3 and T-bet expression but no longer express CD45RA. This transient expression of Foxp3 and CD25 blurs the distinction between Treg and effector T cells. These effector CD4^+^T cells produce IL-2 that promotes polyclonal expansion of tTreg. Activated T effector cells, in the presence of IL-2 and IFN-γ, get further activated to express the transcription factor T-bet and acquire chemokine receptor CXCR3 and produce IFN-γ. In parallel, in the presence of an antigen, IL-2 induces proliferation of resting tTreg that recognize the specific-alloantigen. This activated antigen-specific Treg population (CD4^+^CD25^hi^CD127^lo^CD45RA^-^Foxp3^hi^) is induced to express the receptor for late Th1 cytokines IL-12 and IFN-γ. Further, these activated cells, in the presence of IL-12 or IFN-γ become Th1-like Treg, which express mRNA for Th1 transcription factor T-bet, Th1 cytokine IFN-γ and Th1 chemokine receptor CXCR3. Th1-like Treg continue to suppress and have a much greater potency than resting/naïve Treg. Because they are progeny of tTreg, they have demethylated TSDR and stable Foxp3 expression. These activated memory Treg control immune inflammation in the graft and promote tolerance. They are the mediators of transplant tolerance. In the presence of TGFβ, but not IL-6 or IL-1, naïve T cells can become induced Treg (iTreg) expressing CD25 and Foxp3. These iTreg are unstable as their TSDR is not demethylated. In an inflammatory environment they can revert to activated effector T cells. Whether they can become stable activated/memory Treg is unclear.

CD45RA is the high molecular weight form of CD45 (220kd) and is expressed by non-activated effector and regulatory CD4^+^T cells and other leukocytes. Upon activation both effector and regulatory T cells, but not other leukocytes, splice out one or more exons of CD45 (34). Initially intermediate molecular weight isoforms CD45RB and CD45RC are expressed. Activated T cells and memory T cells express CD45RO, the lowest molecular weight (180kd) form (34, 35). Activated Treg also do not express CD45RA but express CD45RB, RC and RO (27, 35) (**Figure 1**).

Class II MHC, HLA-DR in man, is expressed on activated Treg, but not effector T cells, and is a valuable marker of these cells.

One set of markers that change on activation of Treg is predictable from an early observation that tolerance mediating T cells do not recirculate from blood to lymph (36) but like memory effector T cells reside in tissues and do not recirculate (37). Activated Treg lose expression of CD62L and CCR7, which promote Treg migration into secondary lymphoid organs, and acquire chemokine receptors that promote migration into sites of inflammation (38).

These key markers remain important identifiers of activated, tolerance mediating CD4^+^CD25^+^ Treg. Molecules that differ between resting/naïve Treg and activated Treg are summarized in **Table 1** and described in detail elsewhere (17, 27, 39, 40).

**Table 1:** Markers of Naïve and activated Treg.

Marker	Naive	Activated/Memory
TCR	Polyclonal	Antigen-specific
CD4	+++	+++
CD25	+++	++++
Foxp3	+++	++++
Other Transcription Factors	–	T-bet, RORγT, GATA 3 or Bcl6
CD45RA	++++	–
CD45RB, RC RO	–	++++
CD62L	+++	–
Chemokine receptors	CCR7	CXCR3, CCR8, CCR6, CXCR5
Effector Molecules	CTLA4	CTLA4, CD39, HLA Class II MHC, PD1
CD31- Thymic immigrants	+	–

Five years after CD4^+^CD25^+^T cells were identified to mediate transplant tolerance (22), Sakaguchi et al. identified that CD4^+^CD25^+^T cells produced by the thymus (tTreg) prevent autoimmunity (41). These cells also inhibit allograft rejection (42, 43). They described CD4^+^CD25^+^ T cell that controlled autoimmunity as activated T cells. We described CD4^+^CD25^+^T cells that maintain transplant tolerance as activated T cells and had looked for CD25 expression as they behaved as activated cytokine dependent T cells (22, 26, 44). It is now known that the tTreg described by Sakaguchi et al., are resting cells that have not been activated by antigen in the periphery but have been selected for reactivity to autoantigens in the thymus. They are unlike CD4^+^CD25^+^Treg from animals with transplant tolerance which have been activated in the periphery as part of an immune response to an antigen. tTreg suppress induction of autoimmunity in a non-antigen specific manner (42), whereas activated Treg from transplant tolerant hosts suppress *in vivo* in an alloantigen specific manner (22, 45).

CD4^+^CD25^+^ Treg represent <10% of peripheral CD4^+^T cells and include resting tTreg and activated Treg, induced Treg (iTreg) as described below and activated effector cells. Naive tTreg inhibit antigen-presentation through cytotoxic T-lymphocyte-associated protein 4 (CTLA4) down-regulating the co-stimulatory ligands CD80 and CD86 (46–48). CTLA4 on Treg depletes CD80/86 by trogocytosis, thereby reducing the ability of antigen presenting cells (APC) to activate T cells (49). This process of depletion of CD80/86 on APC also releases free PD-L1 from APC and this further inhibits activated T cells (49). Naïve tTreg are weak at suppressing alloimmune responses (50) and to fully suppress allograft rejection they are required at unsustainably high ratio of 1:1 or 1:2 to CD4^+^CD25^-^ effector T cell (42, 50).

In the humans, CD4^+^CD25^hi^ T cells in blood include the equivalent of tTreg described by Sakaguchi et al. in mice but also identify activated Treg as well as activated CD4^+^ T effector/memory cells. Distinguishing the relative proportion of naïve resting tTreg from activated Treg and activated effector cells is now possible.

CD4^+^CD25^+^Treg express forkhead box protein P3 (Foxp3), a transcription factor that confers regulatory status, including inhibition of IL-2 production and induce expression of CD25 (51). tTreg have demethylation of Treg specific demethylation region (TSDR), which stabilizes expression of Foxp3 (52).

In states of transplant tolerance, the proportion of CD4^+^CD25^+^T cells remains in the usual range of <10% of CD4^+^T cells (53), demonstrating that within this population there are highly potent Treg that suppress rejection even at very low ratio to effector cells. Their phenotype is distinct to tTreg (22, 40, 44). These rare alloantigen-specific Treg are of greatest importance when monitoring transplant recipients for tolerance. This review will examine techniques that may identify activated antigen-specific Treg.

It is apparent other regulatory cells and other mechanisms may contribute to transplant tolerance induction and maintenance including but not limited to CD8^+^Treg, Regulatory B cells, tolerogenic APC and myeloid cells. Exhaustion of effector cell response and partial clonal deletion can also contribute to immune tolerance, these mechanisms are beyond the scope of this review.

## T regulatory cells in transplant tolerance

CD4^+^CD25^+^Foxp3^+^Treg can induce and maintain unresponsiveness to antigens in the absence of clonal deletion (54, 55). Refinement of techniques to monitor subpopulations of Treg may allow more conclusive interrogation of Treg and identify a state of operational tolerance to transplanted tissue and loss of tolerance in autoimmunity.

Within the CD4^+^T cell population, the CD25^+^Foxp3^+^cells are the main regulatory cells. Foxp3 is a transcription factor that induces regulatory function of the lymphocytes. Natural Treg (nTreg), also known as thymus derived Treg (tTreg), are produced in the thymus. These naïve cells and their progeny have stable expression of Foxp3 and CD25 as the Foxp3 TSDR/CNS2 enhancer element is demethylated (52) but demethylation alone is not sufficient to maintain regulatory function (56). In transplant tolerance, hosts have naïve tTreg, as well as activated alloantigen specific Treg.

The evidence that naïve tTreg, CD4^+^CD25^+^Foxp3^+^T cells, can be activated by an allograft to expand alloantigen specific potent Treg has been reviewed elsewhere (17, 40). Much of the literature assumes CD4^+^CD25^+^Foxp3^+^Treg are naive cells and only suppress in a non-antigen specific manner. This is an erroneous assumption.

Induced Treg (iTreg), are peripheral CD4^+^T cells that have been activated by antigen in the presence of TGF-β and IL-2 (57) and the absence of inflammatory cytokines such as IL-1β, as reviewed (58). In the presence of inflammation, especially IL-6, iTreg are unstable and can revert to effector cells, as their Foxp3-TSDR is not demethylated. Studies of CD4^+^CD25^+^FoxP3^+^T cells do not differentiate individual activated tTreg and their progeny, from iTreg.

Some effector/memory CD4^+^T cells also express CD25 and Foxp3 but are not inhibitory (59). Effector CD4^+^CD25^-^Foxp3^-^T cells can be converted to graft protective cells during induction of tolerance in a murine model (60) showing some CD4^+^CD25^+^Foxp3^+^ T cells in tolerant hosts may be induced Treg,

Effector CD4^+^CD25^-^Foxp3^-^ T cells can be induced to have a regulatory function but do not express CD25 or Foxp3, these include Tr1 cells induced by repeated activation of CD4^+^T cells in the presence of IL-10 and antigen (61). Also, Th3 cells which are induced by stimulation with antigen and TGF-β (62). The inhibitory cytokine IL-35 can transform effector cells into regulatory T cells, known as iT_R_-35, which are Foxp3^-^ and suppress by release of IL-35, not IL-10 or TGF−β (63). None of these effector CD4^+^T cells expresses Foxp3 or CD25 and are not identified by the studies covered in this review.

## The adaptive immune response to an allograft

There are multiple pathways of activation of CD4^+^T cells that induce different sets of cytokines (64, 65). This activation is of both effector CD4^+^CD25^-^Foxp3^-^T cells and of CD4^+^CD25^+^Foxp3^+^Treg. The activation of both cell types involves recognition of the same antigen by T cell receptors specific to the alloantigen and cytokines produced by the immune response, mainly effector cells and APC. Thus, effector CD4^+^T cells and Treg are activated by the same cytokines and are induced to express similar effector molecules, cytokines, and transcription factors. Treg can best be envisaged as a T cell with expression of a transcription factor Foxp3 that protects its regulatory functions and stops development of effector cell functions that could damage the graft. tTreg are activated in all immune responses to act as a natural self-regulation to prevent excessive and damaging inflammation.

## The rejection response to an allograft is heterogeneous

Allograft rejection can be mediated by T cells, especially CD4^+^T cells, in the absence of alloantibody, B cells (54, 66, 67) and CD8^+^T cells (68). Activation of CD4^+^T cells is required to help induction of B cells and cytotoxic CD8^+^T cells, making control of CD4^+^T cell activation central to induction of transplant tolerance. In a similar manner, activated effector CD4^+^T cells produce cytokines that promote activation and differentiation of tTreg to alloantigen specific Treg.

Thymocytes and recent thymic emigrants do not effect rejection and inhibit other peripheral T cells (66, 69). Thus, thymic cells act by non-antigen specific suppression, much like tTreg.

The dominant response to allografts is naïve effector CD4^+^ T cell activation driven by antigen and IL-2 to induce Th1 cells that produce IL-2 and express IL-2R including CD25. IL-2 produced by activated T cells has an autocrine/paracrine effect and binds to IL-2R to promote further activation of T cells to Th1 cells producing interferon–gamma (IFN-γ) and tumour necrosis factor alpha (TNF-α) and beta (TNF-β) (70) (**Figure 1**). Th1 cells express the transcription factor T-bet and chemokine receptor CXCR3 (71). Th1 cytokines, IL-12 and IFN-γ activate alloantigen-specific cytotoxic CD8^+^T cells, B cells that produce complement-fixing antibody (72) and macrophages (67). This Th1 inflammation mediates injury to the allograft by induction of expression of Class I and II MHC by graft cells (73) and release of cytotoxic cytokines such as TNF-β.

Th2 cells are both activated by, and produce, Type-2 cytokines. This process is driven by IL-4. Th2 cells produce IL-5, IL-13, and other cytokines. Their expression of CCR8 promotes migration to sites of Th2 inflammation. Th2 cells activated in response to an alloantigen can mediate rejection (45, 74), albeit Th2 cells are not considered the main mediators of rejection.

Th17 cells are induced by antigen in the presence of IL-6, TGF-β and IL-23. They produce IL-17A, IL-17F and IL-22, and express the transcription factor Rorγt and the chemokine receptor CCR6. Depending on the cytokine milieu they can revert to other Th phenotypes (75). The role of Th17 responses in allograft rejection is unclear, as rejection is not associated with a neutrophil infiltrate, which is the hallmark of a Th17 response. In mice with no Th1 response, Th17 cells mediate rapid allograft rejection (76). Th17 associated molecules such as IL-17A and IL-23 are increased in RT patients with acute rejection (77). Increased Th17 relative to Foxp3 cells in renal allograft biopsies indicates a poorer outcome (78) as does increased Th17 cells in blood (79). In operationally tolerant RT recipients Th17 responses were low compared to other RT patients and healthy volunteers (HV) (80). Treg can control Th17 responses in RT patients (81).

T-follicular helper cells (Tfh) are induced by antigen, IL-6, IL-21, and Inducible T-cell Co-stimulator (iCOS). They express the transcription factor Bcl6 and CXCR5 that promotes their migration to B cell areas into germinal centres in secondary lymphoid tissues. They provide help to B cells through CD40L and secretion of IL-21 and IL-4 and induce production of highly specific antibodies (82). Tfh provide help for antibody production in the germinal centres of lymphoid tissues and in transplanted patients, contribute to the donor specific alloantibody response. Circulating Tfh and activated B cells increase during antibody mediated rejection (83–87).

In the absence of IL-6 and an inflammatory response, TGF-β and antigen induce effector CD4^+^T cells to iTreg/pTreg, a regulatory CD25^+^ and Foxp3^+^ phenoype (88). IL-2 contributes to the activation of iTreg by engagement of the IL-2 receptor in the presence of TGF-β (89, 90). IL-2 binding to CD25 enhances proliferation, potency and stability of Foxp3 expression by iTreg (90, 91). TGF-β1 and IL-2 induce iTreg to express Foxp3, and are alloantigen specific in their protection (92). iTreg are unlikely to be induced when there is inflammation from rejection but may develop over time when there is no rejection and inflammation, and IL-6 is not present to prevent iTreg induction (**Figure 1**).

## CD4^+^CD25^+^T cells activation to regulate allograft rejection

IL-2 produced by activated T effector cells in Th1 responses polyclonally expands CD4^+^CD25^+^Foxp3^+^Treg. Murine studies found alloantigen and IL-2 induce expression of receptors for other Th1 cytokines, IFN-γ (31) and IL-12 (29). These IL-2 and alloantigen-induced Treg can be further activated by the same alloantigen and IL-12 and are activated to a Th1-like Treg that expresses IFN-γ and the Th1 transcription factor T-bet but continue to express CD25 and Foxp3 (28). These cells do not produce IL-2, and express the Th1-directing chemokine receptor CXCR3, not CCR7 (**Figure 1**). They suppress naïve alloreactive cells at ratios of <1:1000 (28). IFN-γ also promotes induction of activated antigen-specific Treg (60, 93, 94).

Separate pathways of activation of Treg by antigen and Th2, Th17 and Tfh cytokines respectively induce highly activated Th2-like Treg, Th17-like Treg and follicular T regulatory cells (Tfr Treg) (17, 27). Respectively, they express the lineage transcription factors Gata3, Rorγt, Bcl6 (17) and chemokine receptors CCR8, CCR6, CXCR5 (95, 96).

Activated and antigen-specific Treg migrate to sites of inflammation. Their suppressor mechanisms vary, and some may not yet be described. Naive tTreg may inhibit through cytotoxic T-lymphocyte-associated protein 4 (CTLA4), which down-regulates co-stimulatory ligands CD80 and CD86 on APC. This likely leads to reduced ability of the APCs to activate effector cells. Other inhibitory mechanisms include generation of extracellular adenosine by CD39, granzyme and perforin release leading to cell apoptosis, inhibition of effector cells by CTLA-4, the PD1/PD1 ligand pathways, and production of inhibitory cytokines TGFβ, IL-10 and IL-35 (97–103). Not all described mechanisms are simultaneously employed (104). Some suppression mechanisms may be more dominant than others.

Use of immune checkpoint inhibitors CTLA4 and PD1 to treat malignancy in some RT patients has resulted in a high rate of rejection, indicating a role of Treg and highlighting the importance of CTLA4 and PD-1/PDL-1 pathways in Treg mediated suppression of rejection (105).

## Identification of Treg population in peripheral blood

Treg can be identified by mAb staining of whole blood or peripheral mononuclear cells. Numerous combinations of cell surface and intracellular markers have been used to stain Treg in RT patients, summarised in **Table 2**.

**Table 2:** Markers used to identify T regulatory cells in renal transplantation.

Year	First Author	CD3	CD4	CD25	Foxp3	CD127	CD45R	Chemokine receptor	Activation molecules
**2003**	Salama (106)	–	+	+	–	–	–	–	CD134, iCOS^lo^
**2003**	Game (107)	–	+	+	+	–	–	–	–
**2006**	Louis (108)	–	+	+	+	–	–	–	–
**2006**	Lopez (109)	–	+	+	+	–	–	–	–
**2006**	San Segundo (110)	–	+	+	+	–	–	–	–
**2007**	Bestard (111)	–	+	+	+	–	–	–	–
**2007**	Kreijveld (112)	+	+	+	–	–	–	–	–
**2007**	Noris (113)	+	+	+	–	–	RO	–	–
**2007**	Braudeau (53)	–	+	+	+	–	–	–	–
**2008**	Akl (114)	–	+	+	–	–	–	–	–
**2008**	Bloom (115)	–	+	+	+	–	–	–	CTLA4
**2008**	Bluestone (116)	–	+	+	+	+	–	–	–
**2008**	Daniel (117)	–	+	+	+	+	–	–	HLA-DR, IFN-γ
**2008**	Kreijveld (118)	–	+	+	+	–	–	–	–
**2009**	Hendrikx (119, 120)	–	+	+	–	–	RO	–	–
**2009**	Presser (121)	–	+	+	+	+	–	–	–
**2009**	Kim (122)	–	+	+	–	–	–	–	–
**2009**	Wang (123)	–	+	+	+	–	–	–	–
**2009**	Sewgobind (124)	–	+	+	+	+	RO	CCR7	–
**2010**	Carroll (125)	–	+	–	+	+	–	–	CD69
**2010**	Fourtounas (126)	+	+	+	+	+	RA/RO	–	HLA-DR^hi^
**2010**	Vondran (127)	–	+	+	+	+	–	–	–
**2010**	Sagoo (128)	–	+	+	+	–	–	–	–
**2010**	San Segundo (129)	–	+	+	–	+	RO	–	–
**2011**	Hoerning (130)	–	+	+	+	–	–	CXCR3	–
**2011**	Iwase (131)	–	+	+	+	–	–	–	–
**2011**	Valloton (132)	–	+	+	+	+	–	–	–
**2012**	Hoerning (133)	–	+	+	+	–	–	CXCR3, CCR5	–
**2012**	Krystufkova (134)	–	+	+	+	+	–	–	–
**2012**	Lin (135)	–	+	+	+	–	–	–	–
**2012**	Macedo (136)	–	+	+	+	+	–	–	–
**2012**	Schaier (137)	–	+	+	–	+	RA	–	HLA-DR^hi^
**2012**	Zhao (138)	–	+	+	+	–	RA	–	–
**2013**	Schaier (139)	–	+	+	+	+	–	–	HLA-DR^hi^
**2014**	Bouvy (140)	+	+	+	+	+	RO	–	Helios, CD31 (thymic-derived)
**2014** **2014**	San Segundo (141) Hu (142)	- -	+ +	+ +	+ +	- -	RO -	- CXCR5	CD62L -
**2015**	Krepsova (143)	+	+	+	+	–	–	–	–
**2015**	Braza (144)	+	+	+	+	+	RA	–	CD39, GITR
**2015** **2015**	Wlasiuk (145) Ma (146)	- -	+ +	+ +	+ +	- -	- -	- -	- IFN-γ and IL-17
**2016**	Trojan (147)	+	+	+	+	–	–	CXCR3	CD28, HLA-DR, CTLA-4, Perforin, Fas-L
**2017**	McRae (148)	–	+	+	–	–	–	–	CD39
**2017**	Trojan (149)	+	+	+	+	+	–	–	Helios, IFN-γ
**2018**	Durand (150)	–	+	+	+	+	RA	–	CD39
**2018**	Mathew (151)	+	+	+	+	+	RA	CCR7, CXCR3 CXCR4	Helios
**2019**	Mederacke (152)	–	+	+	+	–	–	–	GARP
**2019**	San Segundo (153)	–	+	+	+	+	–	–	–
**2019**	Krajewska (154)	+	+	+	–	+	–	–	–
**2020**	Roemhild (155)	–	+	+	+	+	–	–	HLA-DR
**2020**	Mirzakhani (156)	–	+	+	+	–	RA	–	–
**2021**	Harden (157)	–	+	+	+	+	–	CCR7	HLA-DR, CD57
**2021**	Shahi (158)	–	+	+	+	–	RA	–	CD31
**2021**	Tamita (159)	–	+	+	–	+	RA	–	–

Earlier studies defined Treg as CD4^+^CD25^+^ (114) (122), however activated effector T cells also express CD25 (160). Thus, early studies on Treg were detecting activated effector cells as well as Treg. Identification of Treg based on CD4 and CD25 expression is not ideal as activated effector CD4^+^CD25^+^T cells promote the rejection response and effect rejection (32). The CD4^+^T cells with higher CD25 expression suppress rejection (161).

Other markers for Treg were subsequently described and these will be discussed here. Each molecule has added precision to the identification of Treg, but all have potential limitations.

Foxp3 identifies Treg (53, 111, 113, 126, 129, 134, 135, 145) and is the transcription factor that promotes the functional CD4 ^+^ Treg. It is an intracellular molecule, not expressed in the cell surface. The detection of Foxp3 requires intracellular staining (162) thus this marker cannot be used to sort out viable Treg. In mice, Foxp3^+^ cells do not always have strong CD25 expression (163). Foxp3 remains the major identifier of Treg, albeit activated effector T cells can transiently express Foxp3.

Treg have demethylated TSDR/CNS2 - the enhancer element for Foxp3. Demethylation of TSDR stabilizes Foxp3 expression in Treg. Demethylated TSDR identifies tTreg and their progeny. Effector lineage CD4^+^T cells and iTreg can transiently express Foxp3 (164) but do not have demethylated TSDR, making Foxp3 expression unstable (165). Detection of demethylated TSDR requires cell DNA, and cannot be used to enrich/sort Treg.

In humans, low expression of the interleukin-7 receptor (CD127) on the surface of cells identifies Foxp3^+^ cells. CD4^+^CD25^hi^CD127^lo^ T cells are highly suppressive *in vitro* (166, 167). The CD4^+^CD25^hi^CD127^lo^ cells are a functional Treg population in renal transplant patients (119, 126, 132, 137, 154, 168, 169).

**Figure 2:**
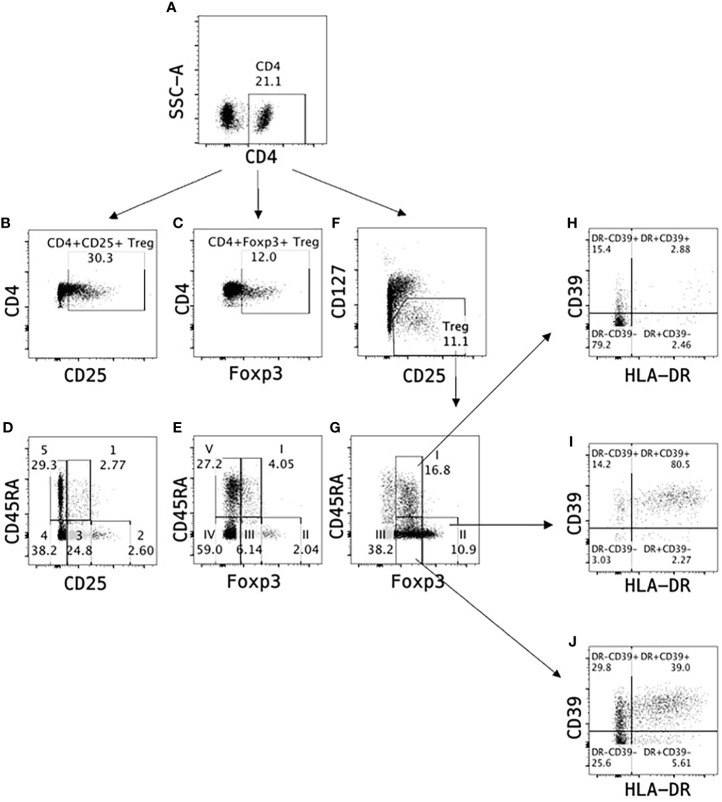
Identification of T regulatory cells in peripheral blood. Treg can be monitored as a proportion of CD4^+^ cells **(A)** in peripheral blood based on expression of CD25 **(B)** or Foxp3 **(C)**. Alternatively, Treg subpopulations can be identified as (1) resting Treg CD4^+^CD25^+^CD45RA^+^ (Population 1 in **D**) or CD4^+^Foxp3^+^CD45RA^+^ (Population I in **E**), activated Treg CD4^+^CD25^hi^CD45RA^-^ (Population 2 in **D**) or CD4^+^Foxp3^hi^CD45RA^-^ (Population II in **E**) and non-suppressive cytokine secreting Treg CD4^+^CD25^+^CD45RA^-^ (Population 3 in **D**) or CD4^+^Foxp3^+^CD45RA^-^ (Population III in **E**). A more reliable identification in human CD4^+^ cell populations is based on CD25 and CD127 expression where Treg are defined as CD4^+^CD25^+^CD127^lo^ **(F)**, which can further be divided into Treg subpopulations I-III based on Foxp3 and CD45RA expression **(G)**. Majority of Treg Population II (>80%) express activation markers HLA-DR and CD39 **(I)** indicating Population II are activated Treg, whereas Population I **(H)** and Population III **(J)** contain only <3% and <40% HLA-DR^+^CD39^+^ Treg respectively.

CD45RA further delineates non-activated Treg (CD45RA^+^) from activated/memory Treg (CD45RA^-^) (35, 137). CD45RA expression identifies three subpopulations of CD4^+^CD25^+^Foxp3^+^CD127^lo^Treg (35, 170), as described by Miyara et al. (35) and illustrated in **Figure 2**. This identifies naïve/resting tTreg as CD4^+^CD25^+^CD45RA^+^CD127^lo^Treg, which are the human equivalent of the Sakaguchi et al’s tTreg in mice. These inhibit activation of an immune response by APC such as dendritic cells.

CD4^+^CD25^hi^FoxP3^hi^CD127^lo^ cells are highly activated Treg and probably include expanded antigen-specific Treg that maintain transplant tolerance. The third population is cytokine secreting CD4^+^CD25^+^FoxP3^+^T cells, some of which can revert to effector T cells. Their full inhibitory capacity as Treg is unclear.

Resting tTreg which are thymic emigrants, also express CD31 (158) but lose it on activation.

Most of the early studies of Treg in renal transplant are limited as they do not distinguish resting tTreg from the potentially more important activated and alloantigen specific Treg. The use of CD25, Foxp3, CD127, CD45RA staining can identify resting tTreg from activated Treg and activated effector lineage cells. Other markers can identify functionally different activated Treg, and these markers are discussed below.

## Changes in Treg over time in renal transplant patients

Absolute Treg and other lymphocyte subsets numbers are significantly reduced immediately post-RT and gradually recover over three to twelve months to pre-transplant levels (119, 122, 129, 131, 154). CD25^+^Treg are identified as early as 3 months post-RT and persist for years (106). A study of 39 stable RT patients (mean 3 years post-transplant) found CD4^+^CD25^hi^ but not CD4^+^CD25^hi^CD127^lo^ were reduced compared to HV (126). Another cross-sectional study of RT patients found no difference in CD4^+^CD25^+^Foxp3^+^Treg counts in patients who were less than and over 5 years post-RT (145). In this latter study, RT patients without a history of cancer post-transplant had similar numbers of Treg to HV, whereas patients with cancer post-transplant had increased CD4^+^CD25^+^Foxp3^+^Treg. A comparison of 30 stable RT patients (mean 8 years post-transplant), 5 hand transplant patients (mean 5 years post-transplant) and 18 HV found that RT patients had fewer CD4^+^CD25^+^CD127^lo^ compared to the other groups (168).

These studies with limited phenotype analysis used absolute counts, which reflect depletion of T cells as a whole. It is known that there is tight control of Treg numbers relative to effector CD4^+^T cells, so Treg do not exceed 10% of peripheral T cells. Thus, many studies report Treg as a proportion of CD4^+^T cells, which is a more meaningful reflection of Treg potential activity. This is particularly the case in therapies that deplete T cells, such as anti-CD25 mAb and anti-thymocyte globulin/anti-lymphocyte globulin ATG/ALG.

## Effect of immunosuppressive therapy on Treg

### Anti-CD25 mAb

The anti-human CD25 mAbs, Basiliximab and Daclizumab, inhibit CD4^+^CD25^+^T effector cells and reduce rejection in RT (171–173). Anti-CD25 mAb therapy depletes CD25^+^ cells or blocks IL-2 binding to CD25 induced on alloantigen activated effector T cells (174). IL-2 is required for generation and maintenance of Treg (175, 176). Anti-CD25 may affect Treg by downregulating CD25 expression by trogocytosis (123, 127, 177, 178). In some studies, anti-CD25 fails to inhibit CD4^+^CD25^+^Treg mediated suppression *in vitro (* 107).

The effect of anti-CD25 mAb on Treg *in vitro* and *in vivo* has been widely studied. When assessing CD25 expression after anti-CD25 mAb therapy, a second fluorochrome-conjugated mAb that does not compete for the CD25 binding site of the therapeutic mAb must be used to avoid an erroneous conclusion of CD25^+^cell depletion (179). In ten publications on Treg after anti-CD25 mAb therapy in RT, four used mAb that are not blocked by the therapeutic mAb (112, 115, 118, 120), two used a mAb that blocked (127, 143) and the others did not describe the clone (116, 123, 138, 180, 181).

Basiliximab (SDZ CHI 621) is a chimeric mouse-human mAb that has high affinity for CD25 and blocks IL-2 binding, thereby inhibiting IL-2 signalling on T cells (181). Basiliximab *in vitro* does not inhibit suppression by Treg (107). Basiliximab has a prolonged action *in vivo* and leads to transient depletion of CD4^+^CD25^+^Treg and CD4^+^Foxp3^+^T cells in blood (115, 116, 138). A six-week reduction of CD4^+^CD25^hi^T cells was observed in RT patients after treatment with Basiliximab, consistent with its duration of action (127, 134). This reduction was associated with a transient rise in CD4^+^CD25^-^CD127^lo^Foxp3^+^ cells, suggesting the mAb down-regulated expression of CD25 without reducing Treg numbers (127). Six months after anti-CD25mAb treatment Treg had recovered (129).

Daclizumab, a humanized mAb to CD25 that blocks IL-2 binding preventing activation of the high affinity IL-2 receptor. Daclizumab lowers CD4^+^CD25^+^Treg in RT patients (106, 115), but functional Treg return to pre-transplant levels by 8 weeks after a single dose (112). Others report Daclizumab reduces CD25 expression without impeding Treg function (120, 123) or depleting CD4^+^Foxp3^+^Treg (120, 121). Whether the depression in CD4^+^CD25^+^Treg by anti-CD25 mAb therapy prevents their ability to induce tolerance is unclear. Other studies found no reduction in Treg (120).

In multiple sclerosis, continued treatment with Daclizumab without concurrent immunosuppression leads to a high rate of spontaneous autoimmunity (182). This suggests anti-CD25 mAb can reduce Treg function over time and allow autoimmune responses in patients not on concurrent immunosuppression that could suppress autoimmune responses.

The evidence is inconclusive whether anti-CD25 mAb therapy inhibits Treg function or impedes induction of immune tolerance. Anti-CD25 mAb are not used in trials of Treg infusion to RT patients.

### Anti-thymocyte globulin/anti-lymphocyte globulin

Immunoglobulin from animals immunised with human thymocytes (ATG) or lymphocytes (ALG) has been used to deplete peripheral lymphocytes of RT patients for over fifty years. Immunomodulatory effects of ATG include depletion of peripheral lymphocytes through complement-dependent lysis, T-cell activation and apoptosis, blocking surface molecules on lymphocytes, and promotion of Treg (183). ATG derived from rabbit, but not horse, expands inhibitory CD4^+^CD25^hi^Foxp3^+^Treg *in vitro* (184), but *in vivo* initially depletes CD4^+^Foxp3^+^CD127^lo^ cells (124).

Induction therapy with ATG in the first 7 days causes a major reduction in all T-cells, including Foxp3^+^ cells (143). ATG allows early preferential proliferation of Treg compared to Basiliximab induction (140) and in both groups recovery of depletion of Treg was due to homeostatic proliferation in the periphery, not thymopoiesis.

Krystufkova et al. prospectively followed 71 RT recipients who received calcineurin inhibitor (CNI)-based triple immunosuppression and induction with either ATG, Basiliximab or no induction antibody (controls) (134). Compared to controls, CD25^+^Foxp3^+^Treg expansion among CD4^+^T cells was observed in the ATG group at all post-transplant time-points after day 14. A significant decrease in Treg frequency and concurrent transient increase of CD4^+^CD25^lo/-^Foxp3^+^T cells were observed in Basiliximab-treated RT 7–60 days post-transplantation. In another prospective study of 20 RT recipients receiving induction with methylprednisolone and ATG, a similar early (day 5) increase of CD4^+^CD25^hi^ Treg was sustained over 24 months (111).

A cross-sectional study of RT over 100 days post-transplant found no difference in CD3^+^CD4^+^CD25^+^Treg between patients who had received ATG, Basiliximab or Daclizumab (117).

### Cytotoxic T-lymphocyte-associated protein 4 fused with immunoglobulin IgG1 Fc

Abatacept and Belatacept are fusion proteins consisting of the extracellular domain of CTLA4 linked to the modified Fc (hinge, CH2, and CH3 domains) portion of human IgG1. Drug binding to CD80 and CD86 blocks the CD28-CD80/CD86 costimulatory pathway required in T cell activation (185).

Belatacept has much higher affinity to CD80 and CD86 compared to Abatacept due to two amino-acid substitutions (186), and is used in kidney transplantation as an alternative to CNI in maintenance immunosuppression (187, 188). A combination of Basiliximab and Belatacept had no effect on CD4^+^CD25^+^Foxp3^+^Treg compared to CNI-treated patients (116).

Binding of CTLA4Ig to CD80 and CD86 also induces dendritic cell production of the immunosuppressive enzyme indoleamine 2,3-dioxygenase (IDO), which depletes local pools of the essential amino acid tryptophan (189, 190). Experimental evidence suggests that tryptophan deprivation sensitises activated T cells to apoptosis prior to cell division and induces a regulatory phenotype in naïve T cells, further contributing to a suppressive environment (191–193).

A study of stable RT recipients receiving either Belatacept or cyclosporine (CSA) (194), has reported no difference in peripheral CD4^+^CD25^+^Foxp3^+^Treg. However, Belatacept-treated patients have significantly increased IDO^+^ peripheral monocytes and increased percentage of Treg on graft biopsies compared to CSA-treated patients. The same group reported reduced inflammatory Th17,Th2 and M1 cells in Belatacept treated compared to CNI treated RT patients and increased Treg and other regulatory cells (195). In an early small study, there was reduced Foxp3 in renal biopsies of Belatacept treated RT patients compared to CNI treated (196). In the blood, Belatacept treated RT had higher regulatory cell populations than CNI treated, and reduced Th17 and Th22 populations (197). In another study, CD4^+^CD25^+^Foxp3^+^ Treg were not different to CNI treated but Th17 cells were depressed (198). Twelve months post RT, Belatacept treated grafts had less Foxp3 than CNI treated grafts, suggesting Belatacept did not induce Treg (199). Combined Belatacept and Basiliximab therapy in RT had similar Treg numbers to CNI controls, albeit there was an early transient depletion of CD4^+^CD25^+^T cells (116).

### Alemtuzumab

This anti-CD52 mAb (Campath 1H) depletes T cells, B cells and monocytes, with recovery of B cells, NK cells and monocytes taking months. Recovery of CD4^+^T cells is slower and full recovery may take more than a year (113). Within the CD4^+^T cells of RT patients treated with Alemtuzumab there is predominance of memory phenotype T cells (CD45RA^-^) (136, 200). The proportion of CD4^+^CD25^+^Foxp3^+^T cells increases for years, compared with a decrease in patients treated with CSA alone or with Basiliximab (115). Patients treated with Alemtuzumab had greater return of Treg if treated with sirolimus rather than CSA (113).

In a trial of Treg therapy, living donor RT recipients treated initially with Alemtuzumab received an infusion of 5x10^9^ expanded recipient’s Treg 60 days after transplant. It was postulated that the infused Treg would suppress allogeneic responses and induce generation of new Treg. One year after transplant, a 5-20-fold increase in the proportion of CD4^+^CD25^+^Foxp3^+^CD127^-^ Treg was observed but the absolute Treg count had returned to normal after 6 months (151). The relative increase in Treg was an effect of Alemtuzumab and *in vitro* expanded Treg therapy. This shows the relative proportion of Treg to effector CD4^+^T cells may change after lymphocyte depletion.

### Calcineurin inhibitors

CSA and Tacrolimus (Tac) act by blocking the TCR-Ag-MHC pathway of T cell activation in both effector T cells and Treg (201, 202). CSA and Tac respectively bind to cyclophiln and FK506 binding protein (FKBP) (203), CNI affect calcium calmodulin preventing its dephosphorylation of the transcription factor NFAT (202, 203). Thereby NFAT cannot translocate to the nucleus and bind to genes that promote transcription of cytokines. CNI thereby inhibits both Treg and effector T cells activation and proliferation (110, 201).

In RT patients on CNI there is a decline in numbers of CD4^+^CD25^+^Treg by 6-8 weeks, which increases by 6-8 months (204). Transcripts of Foxp3 and CCR7 are higher in stable RT patients without CNI therapy compared to those on CNI (205). Ceasing CNI two years post RT allows development of Foxp3 expression and Treg (197). Similarly, in liver transplant, stopping CNI and starting mycophenolate results in an increase in CD4^+^Foxp3^+^Treg (206).

These clinical findings are consistent with CNI inhibiting activation of Treg.

### Mammalian target of rapamycin inhibitors

The activation of CD4^+^CD25^+^Foxp3^+^Treg does not use the mTOR pathway, whereas activation of effector T cells does. Sirolimus and Everolimus bind to FKBP12 which engage and bind to Target of Rapamycin (201). This blocks Signal 3 so cytokines cannot activate cell division (201). mTOR inhibitors do not inhibit calcineurin.

*In vitro*, Treg expansion can be promoted by mTOR inhibitors, which block effector cell activation but permit Treg activation (207).

Treg numbers in RT patients taking sirolimus are up to four times higher than CNI-based regimens (111, 113, 132, 205). In a randomised controlled trial of live donor RT, switch to sirolimus at 2 months increases Treg numbers compared to controls continuing on CNI (208)

CXCR3^+^Treg (Tregs activated by Th1 response) are present in blood of CNI-treated patients, but in lower proportions than in HV (133). Patients treated with mTOR inhibitors have more CXCR3^+^Treg than those treated with CNIs (130).

Following Alemtuzumab therapy, RT patients treated with sirolimus has greater regeneration of Treg than those treated with CSA (113). Conversion from a CNI to Everolimus is associated with a relative increase in CD4^+^CD25^+^Foxp3^+^Treg and reduced effector CD4^+^T cells (209). In RT patients with squamous cell carcinoma, conversion from CNI to sirolimus increases CD4^+^CD127^lo^CD25^hi^Foxp3^+^T cells after 6 months (210).

The clinical observations of Treg combined with the known permissive effects on Treg by mTOR inhibitors, support a role for these inhibitors in tolerance inducing protocols.

## Correlations of Treg with rejection and stable graft function

The utility of Treg as a non-invasive biomarker for rejection has been extensively studied, with mixed results (109). In a comparison of 15 rejection-free living-related RT patients and 15 RT patients with biopsy-proven chronic rejection two years post-transplant, there is a higher absolute number of CD4^+^CD25^hi^Treg in rejection-free recipients (114), but this is not greater than HV. CD4^+^CD25^+^Foxp3^+^T cells appear to be reduced in acute rejection (146). CD4^+^CD25^+^Foxp3^+^T cells in RT are higher than in dialysis patients, but are reduced with acute rejection (142). CD4^+^CD25^+^Foxp3^+^CD45RA^-^Treg are less in patients with rejection than in those without (156). RT patients with antibody-mediated rejection have lower CD4^+^CD25^+^Foxp3^+^Treg than those with stable graft function (158).

In prospective studies, RT patients with chronic rejection have lower Foxp3, CCR7 and granzyme B mRNA, and higher TLR4 and proteasome subunit beta-10 mRNA compared to RT without rejection (131). CD3^+^CD4^+^CD25^+^CD127^lo^Treg count and gene expression of Foxp3 and IL-10 at month 1 and month 3 correlates with long-term graft function (154). RT patients whose absolute peripheral Treg count is above the median of 14.57 cells/mm^3^ at one year post-transplant have better death-censored 5-year graft survival than those with Treg count below the median (92.5% vs 81.4%) (153).

In cross-sectional studies, patients with chronic rejection have lower CD4^+^CD25^+^Foxp3^+^Treg than rejection-free and operationally tolerant RT (53), who have similar Treg to HV (108). Patients with biopsy-proven chronic rejection have significantly fewer CD4^+^CD25^+^ cells and lower Foxp3 transcripts compared to those with stable rejection-free RT, drug-free tolerant RT, RT on minimal immunosuppression and HV (108). The clinically tolerant RT patients have similar Treg levels to HV.

In a large group of RT patients, those with no rejection have higher Foxp3^+^ cells in CD4^+^CD25^+^ compared with those who had rejection, but on multivariate analysis this is not significant (135). There is a positive linear relationship between glomerular filtration rate and the percentage of CD4^+^CD25^+^Foxp3^+^ cells.

In a cross-sectional study of 156 RT patients on the day of renal biopsy, the percentage of CD4^+^CD127^lo^Foxp3^+^Treg within CD4^+^T cells does not differ between the rejecting and non-rejecting (137). The percentage of highly suppressive DR^+^CD45RA^-^ cells is greatly reduced in biopsy-proven rejection (137). Furthermore, suppressive activity of CD4^+^CD25^+^CD127^lo^Treg from RT patients with rejection is significantly reduced compared to non-rejecting and HV (137). Another study also finds suppressive activity of Treg is significantly lower in RT patients with clinically significant borderline rejection on biopsy and acute graft dysfunction, than if there are no histological changes or acute graft dysfunction (139).

RT patients who developed donor specific antibodies after 12 months, have less Treg than those who did not develop alloantibodies (211).

Overall, freedom from rejection is associated with a greater proportion of Treg, suggesting more detailed analysis may identify patients with low risk of rejection and possibly able to have a lower dose of immune-suppressing therapy.

## Correlations of Treg and operational tolerance to an allograft

Operational tolerance is stable renal allograft function in the absence of immunosuppression. Several studies have documented the B and T cell profiles of these patients (212–215) but there is no consensus as to which are important (216). Treg in operationally tolerant RT patients with no immunosuppression for 12 months and a serum creatinine of <150 μmol/L are compared to those with stable RT graft function on immunosuppression over 2 years with serum creatinine of <150 μmol/L, and also to RT patients with chronic rejection, and HV (144). The operationally tolerant group has a higher proportion of CD4^+^T cells with demethylated TSDR, consistent with more Treg. This group also has more activated CD4^+^CD25^hi^CD45RA^-^Treg. These activated Treg have increased expression of CD39 and glucocorticoid-induced TNF-related receptor (GITR). In a later study, it is reported that patients with tolerant RT maintain a physiological memory Treg/CD4^+^CD25^-^ effector ratio (150).

Drug-free tolerant patients have more B and NK cells detectable than RT recipients on immunosuppressive medications or recipients with chronic rejection (128, 213). Tolerant patients also have less activated CD4^+^T cells, including CD4^int^CD25^+^T cells, compared to HV and other transplant patients (128). Whole blood gene expression levels of Foxp3 and α-1,2-mannosidase both correlate with anti-donor immune reactivity after RT (217). Tolerance is associated with a high ratio of peripheral blood Foxp3 to α-1,2-mannosidase expression (128).

## Treg in renal transplant patients with malignancy

In non-transplant patients, tumour infiltration with high numbers of CD4^+^CD25^hi^Foxp3^+^ and CD8^+^CD28^-^T cells are poor prognostic markers (218, 219). In two studies of RT, patients with squamous cell skin cancer have higher CD4^+^CD25^+^Treg than those without cancer, and have lower T cell counts and lower CD8/Foxp3 ratios (125, 135). This limited data suggest too much regulation may increase the risk of malignancy.

## Activated/memory Treg

### CD45RA ^–^/CD45RO^+^

Naïve T effector cells and Treg express the high molecular weight form of common leukocyte antigen (CD45RA), and upon activation express lower molecular weight forms, CD45RB, CD45RC and CD45RO. CD45RA staining of Treg is shown in **Figure 2**. In neonates, the majority of Treg are CD4^+^CD25^+^CD45RA^+^Foxp3^+^T cells and the proportion drops with age as naïve tTreg are activated to Treg that are CD45RA^-^ (220).

In RT patients with operational tolerance, there is an increase in CD4^+^CD45RA^-^Foxp3^hi^ cells that are identified as memory Treg (144, 221). CD4^+^ cells from these patients have a higher level of demethylated TSDR, indicating they are derived from professional tTreg with stable Foxp3 expression. These Treg have higher expression of CD39 and GITR.

In stable RT patients, CD4^+^CD25^+^CD45RO^+^Foxp3^+^CD127^lo^T cells are reduced and the CD4^+^CD25^+^CD45RO^+^Foxp3^-^CD127^hi^ population increased compared to HV (222). The reduction in Treg is greater than in studies that use only CD4^+^CD25^+^ as a Treg marker. The CD4^+^CD25^+^CD45RO^+^Foxp3^-^CD127^hi^T cells from stable renal transplants do not suppress and secrete Th1 cytokines IFNγ and TNF-α. In chronic rejection, there is further increase in CD4^+^CD25^+^CD45RO^+^Foxp3^-^CD127^hi^T cells compared to stable patients, and a complementary reduction in the suppressor CD4^+^CD25^+^CD45RO^+^Foxp3^+^CD127^lo^T cells. The CD4^+^CD25^+^CD45RO^+^Foxp3^-^CD127^+^T cells are found to be allospecific and infiltrate the renal allograft.

Increase in post-transplant CD4^+^CD25^+^CD45RO^+^Foxp3^-^CD127^+^T cells is reported in CNI-treated RT patients but not in those with CNI-free immunosuppression, where activated CD4^+^CD25^+^T cells remained similar tolike HV (132).

Compared to HV, in RT recipients CD25^hi^CD127^-^CD4^+^ T cells are reduced and are lower in a subset of 11 patients who developed donor specific antibodies (159). In this study patients developing anti-donor antibodies, but not those with non-donor anti-HLA antibodies, have more activated CD4^+^CD25^+^CD127^lo^CD45RA^-^ Treg (159). The increased activated Treg may indicate the active immune response to the RT may expand activated Treg in an attempt toto control rejection.

Paradoxically, a multicentre study shows that a high proportion of memory Treg (CD4^+^CD25^hi^CD62L^+^CD45RO^+^ cells) pre-transplant predicts acute rejection in the first year post-RT (141).

Theory on Treg suggests the more activated Treg that are CD45RA^-^ the better the graft outcome. The results to date suggest this may be the case. Increase in activated Treg may indicate a form of tolerance. This hypothesis needs to be tested further with larger studies.

### Treg activated by a Th1 response

Like effector Th1, Treg activated by Th1 cytokines acquire expression of CXCR3, which promotes migration to sites of Th1 inflammation. Th1-activated Treg, like Th1 effector T cells, also lose expression of CCR7 and CD62L, the molecules that promote tTreg migration to secondary lymphoid tissues (223, 224). IP-10 (CXCL-10) attracts CXCR3-expressing cells, both effector and Treg, into the site of graft rejection (225).

Treg activated by Th1 cytokines, in particular IL-12, become Th1-like Treg that express the Th1 transcription factor T-bet as well as Foxp3, and produce IFN-γ but not IL-2 (28, 226). IFN-γ-expressing CD3^+^CD4^+^CD25^+^Treg have been detected in RT patients, especially those with stable graft function (117). Such patients have a similar proportion of IFN-γ^+^ Treg to HV, and the IFN−γ^+^Treg include Helios^+^ and Helios^-^ Treg and methylated TSDR, suggesting a mixture of activated tTreg and induced Treg (149). *In vitro* induction of T-bet^+^IFN-γ^+^Treg is inhibited by methylprednisolone, CSA and high-dose azathioprine and mycophenolate mofetil (227). In long-standing stable RT patients, IFN-γ^+^Treg are associated with high NK cells, stable Foxp3 expression, and expression of Helios and HLADR (147). *In vitro*, CD4^+^CD25^+^Foxp3^+^IFN−γ^+^Treg suppress and induce apoptosis in effector cells (228, 229).

The studies on IFNγ^+^ Treg may identify highly activated Th1-like Treg, which have much greater suppressing capacity. Such Treg activated by an antigen and cytokines produced by a Type I effector T cell response may be major contributors to tolerance induction and important to monitor if tolerance is to be detected.

### HLA-DR-expressing Treg

In the original description of CD4^+^CD25^+^T cells as mediators of transplant tolerance, it was identified the CD4^+^CD25^+^T cells expressed Class II MHC (22). Class II MHC is only expressed by a small subset of peripheral T cells. Studies in multiple sclerosis characterised CD4^+^CD25^+^Foxp3^hi^HLA-DR^+^ Treg, which are highly suppressive (230).

An HLA-DR^hi^CD45RA^-^ subset of Treg is found to be reduced in the first 12 months post-RT but recovers after 12 months (137). This population is higher in RT patients without acute rejection and does not recover in patients with biopsy-proven (including borderline) rejection (139). Treatment with methylprednisolone increases the proportion of highly suppressive HLA-DR^+^Treg (231).

HLA DR expressing Treg may be major mediators of transplant tolerance and further studies are required to establish their usefulness in monitoring for transplant tolerance.

### CD39-expressing Treg

CD39 is an ectonucleotidase that degrades extracellular nucleotides ATP and ADP to AMP. ATP and ADP released from damaged tissue are pro-inflammatory, activating the purinergic P2 receptor (232). CD39 is expressed on some memory CD4^+^CD25^+^Foxp3^+^Treg. CD73 then converts AMP to adenosine, which mediates immune suppression (233–235). Adenosine binds to adenosine A2A receptors on activated Th1 and Th2 cells (236) and induces anergy and promotes Treg (237). CD39 expression on CD4^+^T cells can denote memory (m) Treg (CD4^+^CD25^+^CD45RO^+^CD39^+^) and memory effector cells (CD4^+^CD45RO^+^CD25^-^CD39^+^). The latter have Th17 effector potential, but also produce Th1 and Th2 effector cytokines (97, 238).

Dwyer et al. have found CD4^+^CD25^+^CD39^+^T cells, do not differ between HV, RT and end-stage renal failure patients (97). The proportion of CD39^+^mTreg is reduced in CNI-treated hosts. They find that CD4^+^CD25^+^CD39^+^T cells comprise 1.5% of blood CD4^+^T cells.

In antibody-mediated rejection, there is an increase in CD4^+^CD25^-^CD39^+^T cells, which return to non-rejecting RT patient levels when rejection is resolved. In a longitudinal study, compared to stable RT patients, those with acute T-cell mediated rejection have reduced CD4^+^CD25^+^CD39^+^Treg and CD4^+^CD25^-^CD39^+^T effector cells (148). After one year, RT patients have lower CD4^+^CD25^+^CD39^+^Treg and CD4^+^CD25^-^CD39^+^T effectors compared to HV, however cells from stable RT patients exhibit more potent suppressor capacity.

RT patients with operational tolerance, who have been off immunosuppression for >12 months with stable renal function, have an increased proportion of CD39^+^mTreg compared to those with stable RT on immunosuppression (150, 239). Among CD4^+^T cells, only the CD3^+^CD4^+^CD25^hi^CD45RA^-^Foxp3^hi^ cells are increased. CD39^+^Treg from tolerant hosts degrade ATP to AMP similarly to HV but greater than Treg from RT patients with stable allograft function. Operationally tolerant RT, but not RT patients with stable graft function on immunosuppression, have mTreg to effector T cell ratios that are increased, and similar to HV.

CD39, along with HLA DR expressing Treg, appear to be markers of activated Treg. CD39 plays a role in mediating suppression by Treg. Further detailed studies are required to establish their role. One issue is immune response to non-transplant antigens may induce activation of Treg to express CD39, thus there may be a background incidence of CD39 expressing cells not related to the immune response to the allograft.

### T follicular regulatory cells in renal transplant

The nature of Tfr Treg has recently been reviewed (240–242). Tfr are a small subset of CD4^+^T cells that control immune responses in lymphoid follicles and germinal centres. Tfr control germinal B cell activation (243).

Tfr are CD4^+^CD25^+^Foxp3^+^T cells that can be identified by their high expression of CXCR5, PD1 and iCOS. Tfh cells have intermediate expression of CXCR5 and PD1. CXCR5 promote migration to germinal centres by CXCL13 released by germinal centre dendritic cells (240, 242). Tfr inhibit through production of IL-10 and PD1 (244). Other regulatory cells, such as tTreg, regulatory B cells can inhibit alloantibody production (245). In a murine model, while Tfh cells are required for production of alloantibody, absence of Tfr worsenes alloantibody production and rejection (246).

Niu et al. while examining Tfr in blood of 5-7 year old RT patient’s blood, have found no difference in Tfh cells but reduced Tfr cells in RT compared to HV (244), Prior alemtuzumab therapy but no steroid or IVIg therapy has no effect on Tfr (244). Yan et al. have reported lower Tfr cells in RT recipients with chronic allograft dysfunction than in RT patients with stable graft function (247).

Effective induction of Tfr-Treg may prevent donor specific antibody responses - a major cause of graft loss. Their potential as a therapy has been reviewed (248).

## Treg in renal biopsies and urine

Veronese et al. have described increased Foxp3^+^ cells in acute cellular rejection than in antibody mediated rejection (249). The majority of cells are CD4^+^ and are located in tubules, which may account for the high levels of Foxp3 in urine during rejection. Higher Foxp3^+^ cell numbers correlate with a worse prognosis (249), Others have reported early infiltration of Foxp3^+^ cells in renal biopsies taken early after transplant, especially if there is acute cellular rejection (250). Examining the ratio of Foxp3 to granzyme B mRNA expression, Grimbert et al. found it is greater in borderline rejection than in acute cellular rejection of RT (251), Overall it has been suggested that expression of Foxp3 in biopsies may predict a better outcome (252).

Bestard et al. have found that in 6-month protocol RT biopsies with borderline rejection, those stained for Foxp3^+^ cells are from patients who have better graft function at 2-, 3- and 5-years post-RT than those with no staining (253, 254). Foxp3^+^Treg/CD3^+^T cell ratio also correlates with better graft function at 2 years. Treg predominance over cytotoxic T cells in surveillance renal biopsies predicts good outcome, whereas cytotoxic T cell predominance is associated with acute cellular rejection (255). In acute T cell-mediated rejection biopsies, predominance of Foxp3^+^ cells over Th17 leads to better graft survival at 1 and 5 years (78). These studies suggest that the proportion of Treg within the T-cell infiltrate is relevant to facilitating renal engraftment.

Bunnag et al. found expression of Foxp3 is higher in biopsies with both cellular and antibody-mediated rejection than those without rejection (256). Chronic antibody-mediated rejection is also associated with increased Foxp3 and infiltration of Foxp3^+^ cells. It is proposed that Foxp3 expression is a time-dependent feature of inflammatory infiltrates, and that entry of Foxp3^+^ cells may stabilise inflammation or eliminate it.

In a study of urinary Foxp3 mRNA and other T cell markers at the time of renal biopsy (257), the mean ratio of Foxp3 mRNA to 18S ribosomal RNA copies is higher in urine from RT patients with acute rejection (n=36) than those with chronic allograft nephropathy (n=18) or normal biopsies (n=29). Foxp3 mRNA levels are inversely correlated with serum creatinine levels in the acute rejection group only. Reversal of acute rejection can be predicted with high sensitivity and specificity by use of an optimal cut off for Foxp3 mRNA. Low urinary Foxp3 mRNA levels identify subjects at risk of graft failure within six months after the episode of acute rejection. T cell markers, including CD25, CD3e, perforin, and 18S rRNA are predictive of reversal of acute rejection or graft failure.

These findings are confirmed in a large multicentre study of 4300 urine samples and 485 RT recipients in the first-year post RT. mRNA expression for CD3e, IP-10 and 18S rRNA is used to diagnose and prognosticate acute rejection (258). This group at a single centre studied 3599 urines from 480 RT recipients, confirming that mRNA for Foxp3, CD25, CD3e and perforin can diagnose acute cellular rejection and the reversibility of the rejection (259).

These studies are consistent with the inflammation of a rejection response attracting Treg into the graft to control rejection. In the absence of inflammation from rejection, there is nothing to attract migration of Treg to the graft. Rejection will attract Treg, so it is not surprising they are present during rejection both in the graft and in urine. The presence of Foxp3^+^Treg may be slowing rejection and contribute to its reversal with a better outcome than when Foxp3^+^ cell number is low.

## Regulatory T cells as therapy for kidney transplantation

The therapeutic potential of ex vivo expanded Treg is explored in a number of studies (151, 155, 157, 260–265), and has been reviewed elsewhere (266–268). Their use heightens the need for better methods of monitoring Treg subsets as a potential diagnostic test for tolerance. To date, studies of expanded naïve Treg therapy has been shown to be safe and cause no harm to RT function. More specialized alloantigen specific Treg, such as Tfr, have been prosed as therapy in RT (248)

## Discussion

This review makes the case that regulatory cells, especially CD4^+^CD25^+^Foxp3^+^T cells, can control transplant rejection and induce a state of operational tolerance where rejection does not occur. In animal models of operational tolerance, second grafts from the same donor strain are not rejected but third party grafts are rejected (269). Thus, a state of specific unresponsiveness develops yet the hosts lymphocyte are not clonally deleted (12) and *in vitro* can respond to specific donor and third-party alloantigen (18, 23, 270).

Human RT recipients can develop operational tolerance, but most patients reject the graft if nonspecific immunosuppression is stopped. Studies on RT patients with operational tolerance have not described a precise mechanism. Most reports identify changes in B cells (213–216). The changes in B cells were inconsistent between studies, however. The potential of regulatory B cells to induce transplant tolerance is not established, but these cells may play a role.

Increased numbers of CD45RA^-^Foxp3^hi^ Treg have been identified in RT patients with operational tolerance (144, 221). This population of CD45RA^-^Foxp3^hi^ Treg is low in RT patients with acute rejection (206). Miyara et al. identified these as fully activated Treg (35) and this is likely the population containing alloantigen specific Treg with great potency at regulation. There is considerable evidence that activated alloantigen specific Treg can induce operational transplant tolerance, but our knowledge of these cells compared to tTreg is very limited. These activated Treg should be the focus of future research to elucidate methods to promote activated alloantigen specific Treg and to monitor them.

Much can be learnt from murine models, and we will summarize some of these issues below.

The tolerant state is maintained by Treg entering the graft and controlling rejection in the graft (271). Thus, there should be a focus on Treg expressing chemokine receptors that promote migration to sites of inflammation such as CXCR3 and CCR6. Treg expressing these chemokine receptors are found in the CD45RA^-^Treg, especially those with high CD25 and Foxp3 expression.

A major underappreciated issue is the differences in cytokine requirement for T cells’ continued survival and function, particularly *in vitro* and especially in reference to naïve tTreg and antigen activated Treg. In early studies, lymphocytes were found to die ex vivo and were considered to have no function (272). To survive *in vitro*, all lymphocytes including Treg need specific cytokines. Effector lineage T cells express CD127 (the IL-7 receptor) and IL-7 is required to stimulate their survival as does IL-6 and IL-4 (273). After activation, effector T cells become dependent on other cytokines such as IL-2, IL-4, IL-12, IL-15 (274).

CD4^+^CD25^+^Foxp3^+^Treg have low or no expression of CD127 (275) and their survival is dependent on IL-2 not IL-7. When activated by antigen and IL-2, Treg become dependent on IFN-γ (29, 44)and IL-12 not IL-2 (28). Activated and alloantigen-specific Treg require continued stimulation by specific antigen and cytokines other than IL-2. *In vitro* without these two requirements, activated antigen specific Treg will not survive. This could lead to a false impression that activated antigen-specific Treg do not exist and have no function, much as was described in the early experiments in lymphocytes *in vitro* (272).

Another major area to consider is that the CD4^+^CD25^+^Foxp3^+^T cell population is not just resting/naïve tTreg but is very heterogeneous. It contains a large number of resting naïve tTreg subpopulation that act in a non-specific manner to inhibit activation of immune responses especially those to autoantigen. These cells are depleted with age (220) but this subpopulation of naïve/resting Treg is still present after renal transplant. This subpopulation is probably not directly relevant when transplant tolerance is established, but during induction of tolerance is the source of naïve Treg for activation by alloantigen and cytokines from the activated effector lymphocytes (17, 39, 40, 276).

The population of greatest interest is the CD45RA^-^ subset of CD4^+^CD25^+^Foxp3^+^T cell, which may include both antigen activated effector T cells and antigen activated Treg. According to Miyara (35), it is these cells with high expression of CD25 and Foxp3 that are highly activated Treg. This population is increased in RT patients with operational tolerance (144, 221) and is potentially the most important. However, some cells of this subset of CD4^+^CD25^+^Foxp3^+^CD45RA^-^T cells may have been activated to other antigens such as autoantigens. In HV, this population represents about 10% of CD4^+^CD25^+^Foxp3^+^T cells, and is increased in diseases such as multiple sclerosis (158). The relevant alloantigen specific Treg in this subpopulation may be rare in the circulation.

Activated Treg migrate to tissue and may accumulate in the graft where there is specific alloantigen and activated T effector cells, that produce the cytokines required for their survival and propagation. Cytokines such as IFN-γ, IL-12 and IL-5 are produced in a fully activated T cells, after they mature and stop producing IL-2 and IL-4. Identification of activated Treg within the graft may reveal an increased number in operational tolerance.

Currently there is no immune monitoring test that determines whether a tolerant state exists so safe withdrawal of immunosuppression can occur. There is no standardised method of estimating Treg (**Table 2**) as early studies counted CD4^+^CD25^+^T cells and later CD4^+^CD25^+^Foxp3^+^ T cells (**Table 1**), unwittingly including activated effector CD4^+^T cells.

Now human Treg should be identified more specifically as CD4^+^CD25^+^Foxp3^+^CD127^lo^ T cells (220, 275). Treg are depleted in the early stages following RT then recover to levels of HV. Operational tolerance in animal models (44) and patients (144, 221) is not associated with marked increases in CD4^+^CD25^+^Foxp3^+^Treg in the circulation and peripheral lymphoid tissues, albeit there are increased proportions of Treg to effector CD4^+^T cells in many studies.

Staining CD45RA, CD4, CD25 and CD127 identifies three Treg, and two effector T cell sub-populations (35) (**Figure 2**). CD4^+^CD45RA^+^CD25^+^Foxp3^+^CD127^lo^Treg are resting thymus-derived cells, known as either nTreg or tTreg that prevent autoimmunity (41). They inhibit naïve effector cell activation by inhibiting antigen presentation by dendritic cells. They only suppress rejection at unphysiologically high ratios to naïve effector cells (42), levels not achieved post-transplant. In chronic inflammation they are depleted (170).

tTreg can be activated by alloantigen and key cytokines (40) to become alloantigen-specific Treg that suppress rejection at low ratios to effector cells (28, 29). CD4^+^CD45RA^-^Foxp3^hi^CD25^hi^ cells are activated Treg, and do not include recently activated CD4^+^CD45RA^-^Foxp3^+^CD25^+^ effector T cells that can produce inflammatory cytokines such as IFN-γ and IL-17 (35). CD4^+^CD45RA^-^Foxp3^hi^CD25^hi^ cells are increased in operationally tolerant patients (144, 221).

When using lack of CD45RA as a marker of activated cells, focus should be on the CD45RA^-^Foxp3^hi^ population (170). Activated Treg that mediate transplant tolerance do not recirculate from blood to lymph and migrate into tissue (36). They do not express CD62L or CCR7. They express other chemokine receptors including CXCR3 for Th1-activated Treg, CCR8 for Th2-activated Treg and CCR6 for Th17-activated Treg (95, 96). Some activated Treg express two chemokine receptors (170). These chemokine receptors direct migration of activated Treg into inflamed tissue where they inhibit inflammation. Th1-like Treg can be identified by their expression of IFN-γ (117). Th1-like Treg are the most potent antigen-specific Treg defined (28). Tfr cells were depleted in RT patients destined to develop chronic rejection (277) and in RT patients with long standing immunosuppression, particularly those that received Alemtuzumab (248). More detailed examination of Treg subpopulations in RT patients is required.

Activated Treg also express molecules that mediate their suppressive effects, including CD39/CD73, IL-35, HLA-DR, CTLA4 and PD1. In a recent study with CyTOF (cytometry by time of flight), over one hundred mAb have been used to segregate subpopulations of lymphocytes (157). Activated/memory Treg expressing these molecules may indicate tolerance and can be assayed.

An area that the current research does not adequately consider is that our early murine studies have identified that alloantigen-specific CD4^+^Treg do not survive when stimulated with specific alloantigen unless key cytokines are available (24, 28, 29, 44). Activated Treg express receptors for many cytokines not normally associated with their function, such as IFN-γ, IL-12, IL-5 (44). Further, CD4^+^CD25^+^Treg from tolerant hosts do not proliferate when stimulated by specific donor alloantigen unless cultures are supplemented with key cytokines (44). This is a critical understanding to have when designing assays that detect alloantigen specific Treg. Ex vivo they rapidly die even if stimulated with specific alloantigen. This is the observation that led us to examine if the tolerance transferring CD4^+^T cell was activated and expressed the activation marker CD25, the IL-2 receptor.

Pardoxically, IL-2 cannot alone fully sustain activated alloantigen specific Treg (44). In studies on enriched CD4^+^CD25^+^T Cells from animals tolerant to an allografts, their *in vitro* proliferation to specific donor is at background and less than to self (44). Adding either IL-2 or IL-4 to culture increases these cells’ proliferation to self, specific donor and third party stimulator cells (44). Thus, there is an antigen nonspecific response promoted by IL-2 (28, 29) and IL-4 (29).

We screened the effects of other cytokines in these cultures and identified three that promote CD4^+^CD25^+^T cells to specific donor but not to third party. Cytokines shown to promote proliferation and survival of activated Treg are produced later during the activation of effector CD4^+^ T cells including IFN-γ, IL-12 and IL-5 (44). Other cytokines such as TGF-β. IL-10 and IL-13 do not promote proliferation of CD4^+^CD25^+^T cells from tolerant animals (44). The cytokines identified to support antigen-specific Treg are produced late in Th1 or Th2 responses, after the early cytokine production, mainly IL-2 or IL-4 has waned. The role of IFN-γ (25) and IL-5 (24) in sustaining tolerance transferring CD4^+^T cells in short-term cultures is confirmed.

We have started to extend these findings using human CD4^+^CD25^+^T cells, and shown culture of normal tTreg cells with an alloantigen and IL-4 induces expression of IL-5 receptor alpha (29). Others found IFN−γ critical for activation of CD4^+^CD25^+^Treg in murine (278) and human (279, 280) studies. IL-12 induces IFN-γ in Treg but only if there is no IL-2 (28, 281). Further, treatment of transplanted hosts with IL-12 (282) or IL-5 (283) delays graft rejection. Taken together, these findings suggest the inflammatory cytokines IL-5, IFN-γ and IL-12 also can inhibit rejection, in part by activating antigen specific Treg.

The immediate challenge in this field is to develop an assay to identify alloantigen-specific Treg that are essential and sufficient to achieve operational tolerance in RT patients. First, this effort will require a better understanding of the stimuli and cytokines that promote activation and expansion of antigen specific Treg. Key contenders are IFN-γ, IL-12 and IL-5. Second, any assay needs to consider the short life span of activated Treg ex vivo and their dependence on specific antigen stimulation and cytokines other than IL-2. Third, to explore the possibility that IL-2 itself may turn off activated Treg. Fourth, to examine if activated Treg turn off activated effector cells, and do not necessarily inhibit APC, as occurs with tTreg. Thus, current assays used to measure suppression by tTreg, may not identify the inhibitory potential of activated Treg.

Well defined assays need to be developed that may identify states of operational tolerance and guide future research into effective interventions to achieve operational tolerance in RT patients. In future studies, consideration also needs to be given to other mechanisms of transplant tolerance, as CD4^+^CD25^+^CD127^lo^Foxp3^+^Treg may only be one of several key mechanisms.

## Author contributions

JC, BZ, JD initiated the manuscript. MS, JW, SS, NV and SH provided critical feedback and BH edited and supervised the work. All authors contributed to the article and approved the submitted version.

## Conflict of interest

The authors declare that the research was conducted in the absence of any commercial or financial relationships that could be construed as a potential conflict of interest.

## Publisher’s note

All claims expressed in this article are solely those of the authors and do not necessarily represent those of their affiliated organizations, or those of the publisher, the editors and the reviewers. Any product that may be evaluated in this article, or claim that may be made by its manufacturer, is not guaranteed or endorsed by the publisher.
